# Supplementary material for: Identifying mRNAs Residing in Myelinating Oligodendrocyte Processes as a Basis for Understanding Internode Autonomy
Source: Life (Basel). 2023 Apr 4;13(4):945. doi: 10.3390/life13040945 (PMC10142070; doi:10.3390/life13040945)
Supplement: Supplementary file 1 [file life-13-00945-s001.zip › life-2270072-supplementary.pdf]

Supplementary data

Table S1. Real-time PCR primers used in this study.

mRNA	Acc. #	Forward primer	Reverse primer	Size
MBP	K00512	CACAGAGACACGGGCATCC	CTGGGTTTTCATCTTGGGTCC	154
MOBP-81A	X87900	AACGCTGTGCATGTGTTAGC	AACGCTGTGCATGTGTTAGC	130
PLP1	NM_030990	GTGTTTCTTTGGAGTGGCAC	GGCCCCATAAAGGAAGAAGA	181
MOBP-81B	X89637	GGGAATGGGTCTTTTGGTTT	AGAGAGGGGAATTGGAAGGA	164
CNP	M18630	GTCTRGACGAGGACCTGGCT	TAGTCGCCACGCYGTCTTG	161
UGT8A	NM_019276	CCACCTCAGATGCTTTCCTG	GTTGCCAACCATCATGTCAC	122
MOG	NM_022668	GCAGGTCYCTGTAGGCCTTG	GGGCACYCTCAGGAAGTGAG	115
SLC44A1	NM_001033852.1	CTGCTGCAGCTCCGCCTC	GTCTGTGCAGCTACGGTCCT	76
CSPG4	NM_031022	GACCCTCGTCACCTGCTCTA	ATTCCCAGCATTCACCTCAG	126
PDGFRA	NM_012802	GGTCTTATGGCGTTCTGCTC	CCATCCTGTATCCGCTCTTG	111
NEFL	NM_031783	GCCGAAGAGTGGTTCAAGAG	TGTCTGCATTCTGCTTGTCC	150
TUBB3	NM_139254	CTCCCGGGTTAAAGTCCTTC	CAGGCCTGGAGCCATAATAA	127
AQP4	NM_012825	CGGTTCATGGAAACCTCACT	TCAGTCCGTTTGGAATCACA	106
GFAP	NM_017009	CCTGCCAGGGTRGACTTCTC	TGTAGCTAGCAAAGCGGTCA	112
APOD	NM_012777	CGGTGCAAGAGAATTTCGAT	GGACGCAGCTCCTTGTTTAG	154
DYNC1LI2	NM_145772	CTGCTGGGTGARGATGGAG	CCTTTGTGGTAYAGATCTCC	176
ENPP2	NM_012848	TCCCCACCCACTACTACAGC	AAGTGGGCCAGAACTACCAC	187
GSN	NM_001004080	GGTCTTTGTCTGGGTTGGAA	AAAGGAAGGAGGCTCAAAGC	151
KIF1A	XM_343630	GAGGATGAAGTCCAATGGAC	TAGCTCCAGACTGATGGCR	150
LPAR1	NM_053936	ATTTCACAGCCCCAGTTCAC	GTTGACGTARATTGCCACCA	201
MAPK8IP1	NM_053777	CTGAAGACAGGGGAGCAGAC	ATTCGATCTCGATGGGAGTG	208
NDRG1	NM_133583	ATGCCGTGGTGGAATGTAAT	CGAGACAGGCGAGTCATACA	175
PADI2	NM_017226	GAACCTGGTGGAGAAAACCA	GCACTACTGGGAAGCCTTTG	131
SH3GL3	AF009604	GGTGAGGCCCTGAAACTCA	CCTCGGGGATCTTACCYAC	190
TPPP	XM_341746	CCAAGGCTGCTAAGAGGTTG	CTTGCTGAAGACGATGTCCA	417
TRAK2	NM_163550	GATGTCAGAGGAGGCTCACC	AGTGCACTGAGCACACACCT	151
TRP53INP2	XM_00106397	GCCTTTGTCTCTGAGGAGGA	ACAAACCAGCTCTCGTCCAT	149
GAPDH	NM_017008	TGCCACTCAGAAGACTGTGG	TTCAGCTCTGGGATGACCTT	129

Legend: Primer 3 (https://bioinfo.ut.ee/primer3-0.4.0/) was used to select PCR primers to each mRNA. Each primer pair was analyzed with conventional PCR with cDNA prepared from P60 rat cerebral cortex total RNA as template to ensure that single appropriately sized products were obtained. Knowing that the primers amplified the correct mRNA specific products, they were used in the RT-qPCR analyses. Color coding used throughout reference mRNAs located in oligodendrocyte processes (MSAS/OL-P),

oligodendrocyte soma (OL-S), newly-formed oligodendrocytes (NFO), oligodendrocyte precursor cells (OPC), neurons (NE), astrocytes (AST). Putative *MSAS* mRNAs are listed as is *GADPH* used to normalize results.

Table S2. Expression patterns of cell-type specific mRNAs – MBRS data.

#	Gene	OPC	NFO	MO	NE	AST	MG	EP	%MO/CT	%NFO	%OPC	%OL	MO/NFO
MO1	*MBP*	271	**26708**	**95398**	12	2	175	1	**77.8**	**21.8**	**0.2**	**99.8**	**3.6**
MO2	*MOBP*	8	**1214**	**2543**	0	0	3	0	**67.5**	**32.2**	**0.1**	**99.9**	**2.1**
MO3	*MAL*	4	**761**	**2574**	0	1	1	26	**76.4**	**22.6**	**0.1**	**99.2**	**3.4**
NFO1	*GPR17*	**441**	**930**	67	6	0	1	0	4.6	**64.3**	**30.5**	**99.5**	**0.1**
NFO2	*MYRF*	8	**178**	**118**	1	0	0	0	**38.7**	**58.3**	2.6	**99.6**	**0.7**
OPC1	*PDGFRA*	**596**	**80**	1	9	0	1	28	**0.6**	**11.1**	**83.5**	**94.7**	--
OPC2	*CSPG4*	**117**	**23**	0	9	1	0	4	**0.2**	**15.0**	**76.0**	**91.1**	--
NE1	*STMN2*	9	2	6	387	3	6	2	92.3*	--	--	--	--
NE2	*TUBB3*	14	6	17	339	2	5	4	87.6*	--	--	--	--
AST1	*AQP4*	11	1	11	12	318	0	2	91.9*	--	--	--	--
AST2	*ALDH1L1*	6	0	3	8	145	4	2	86.4*	--	--	--	--
MG1	*C1QA*	2	2	96	7	21	3988	0	96.9*	--	--	--	--
EP1	*CLDN5*	4	0	2	8	11	11	3437	98.9*	--	--	--	--

*Represent percentage expression by NE (*), AST (*), MG (*) and EP (*).

Legend: Cell-type-specific expression patterns of mRNAs highly expressed by myelinating oligodendrocytes (MO1-MO3), newly formed oligodendrocytes (NFO1-NFO2), oligodendrocyte precursor cells (OPC1-OPC2), neuron (NE1-NE2), astrocyte (AST1-AST2), microglia (MG1) and ependymal cell (EP1). Values in Fragments Per Kilobase of transcript per Million mapped reads (FPKM). Maximum expression values plus those falling within 10% of the maximum value are in bold and colored based on cell type. Percentages expressed by MO/CT (%MO/CT), NFO (%NFO), OPC (%OPC) and oligodendrocyte lineage (%OL, %OPC+%NFO+%MO) are given in marked columns as well as MO/NFO for MOs and NFOs. These mRNAs were used to identify cell-type-specific expression patterns in AMBA and AMSCA images (Figure 3).

Table S3. Links of cell-type specific mRNAs - AMBA, AMSCA and ADMBA data.

#	mRNA	Br sagittal	Br coronal	SC – P56	SC – P4	Dev Brain
MO1	*MBP*	79632288	12202838	100001620[wm]	100001621[wm]	++
MO2	*MOBP*[1]	69174034	- -	100017643[wm]	100017644[wm]	++
MO3	*MAL*	386243	- -	100006019[wm]	100006018[wm]	- -
NFO1	*GPR17*	73513814	74641313	100032104[wm]	100032105[wm]	- -
NFO2	*MYRF*[2]	70295805	- -	100026205[wm]	100026206[wm]	- -
OPC1	*PDGFRA*	79354743	77280309	100003956	100003955	++
OPC2	*CSPG4*	69608160	- -	100020669	100020670	- -
NE1	*STMN2*	68632984	74819695	100013852	100013853	- -
NE2	*TUBB3*	68196914	- -	100013057	100013058	++
AST1	*AQP4*[3]	109	108	100002844[ra]	100002845[ra]	++
AST2	*ALDH1L1*	1725	1724	100001676	100001677[vdra]	- -
MG1	*C1QA*[4]	68191456	79591671	100015379[lmgm]	100018950	++
EP1	*CLDN5*	79360268	79490120	100002146[vl]	100002147[vl]	++

[1]*MOBP* mRNA – additional sagittal section: 769201510, [2]*MYRF* mRNA – two additional sagittal sections (71161357, 71764437), [3]*AQP4* mRNA – additional sagittal (77454660) and coronal (79556722) sections, and [4]*C1QA* mRNA – additional sagittal section (77923147).

Legend: Links of the three MO-, two NFO-, two OPC-, two neuron-, two astrocyte-, one microglia- and one ependymal cell mRNA used to obtain AMBA and AMSCA images examined in Figure 3 are given. If images were present in the Allen Developing Mouse Brain Atlas (ADMBA), they were indicated (++). In the AMSCA data sets where an expression category is assigned, the category is included as a superscript following the accession number. Categories: cc – central canal, gm – gray matter, ra – white matter and radially arrayed white matter, gw – gray matter and white matter, lm – lamina 1-x and gray matter, rv – radial arrayed and vascular like in gray and white matter, vd – ventral-dorsal midline in gray matter, vl – vascular-like in gray and wm – white matter.

Table S4. Links to *MSAS* mRNAs – AMBA, AMSCA and AMDMB data.

#	mRNA	Brain - sagittal	Brain - coronal	SC – P56	SC – P4	Dev
MSAS1	*LPAR1*[1]	75749420	79556595	100037166[wm]	100037167[wm]	--
MSAS2	*TRP53INP2*	70192081	--	100024314	100024315	--
MSAS3	*TRAK2*[2]	75990672	--	100051344	100051345	--
MSAS4	*TPPP*[3]	69407355	74724775	100018955[wm]	100018956[wm]	--
MSAS5	*SH3GL3*	79632219	--	100002780 (ne)	100002781	--
MSAS6	*GSN*	68444642	72081395	100011054[wm]	100011055 [wm]	--
MSAS7	*APOD*	71380893	--	100020110[wmvl]	100020111[wmvl]	++
MSAS8	*MAPK8IP1*	68148737	--	100022617	100022618	--
MSAS9	*KIF1A*	69735759	--	100030717	100030718	--
MSAS10	*ENPP2*[4]	532631	70613967	100001398[wm]	100001399[wm]	--
MSAS11	*NDRG1*	70429303	--	100039463[wm]	100039464[wmlmgm]	--
MSAS12	*DYNC1LI2*	69627027	--	100022042	100022043	--
MSAS13	*PADI2*	69013438	--	--	--	--
MSAS14	*CAR2*	69014434	72103831	100016060[wm]	100016061[wm]	--
MSAS15	*CRYAB*	72182998	74357575	100008586[wm]	100008585	--

[1]*LPAR1* is listed as *EDG2*, an additional sagittal image series - 79360278, [2]*TPPP* is listed in ASCA as 2900041A09Rik,[3]*TRAK2* mRNA – an additional P56 spinal cord image series: 100039894, [4]*ENPP2* mRNA – an additional brain coronal image series - 77413700

Legend – *MSAS* mRNAs are ordered based on enrichment in myelin vesicles and are color coded according to enrichments. MSAS1 – MSAS5 were enriched at levels suggesting locations in MSAS. MSAS6 – MSAS9 were enriched at levels suggesting locations of oligodendrocyte somata. MSAS10 – MSAS11 were not enriched in myelin (Figure 1). MSAS12 – MSAS13 were not present at levels sufficient to determine enrichment. MSAS14 – MSAS15 were not examined. All *MSAS* mRNAs were obtained from the AMBA (https://mouse.brain-map.org/) and AMSCA (http://mousespinal.brain-map.org/) databases. With the exception of *APOD* mRNA, *MSAS* mRNAs are not found in the Allen Developing Mouse Brain Atlas (http://developingmouse.brain-map.org). Hyperlinks to available data sets are shown. Expression categories from spinal cord image sets as indicated in Table 4S. *SH3GL3* mRNA is not expressed (ne) in P56 spinal cord.
